# Perseveration Found in a Human Drawing Task: Six-Fingered Hands Drawn by Patients with Right Anterior Insula and Operculum Damage

**DOI:** 10.1155/2014/405726

**Published:** 2014-04-30

**Authors:** Chiharu Niki, Takashi Maruyama, Yoshihiro Muragaki, Takatsune Kumada

**Affiliations:** ^1^Institute of Advanced Biomedical Engineering and Science, Tokyo Women's Medical University, 8-1 Kawada-cho, Shinjuku-ku, Tokyo 162-8666, Japan; ^2^Department of Neurosurgery, Tokyo Women's Medical University, Tokyo, Japan; ^3^Graduate School of Informatics, Kyoto University, Kyoto, Japan

## Abstract

*Background*. Perseveration has been observed in a number of behavioural contexts, including speaking, writing, and drawing. However, no previous report describes patients who show perseveration only for drawing a human figure. *Objective*. The present report describes a group of patients who show body awareness-related cognitive impairment during a human figure drawing task, a different presentation from previously described neuropsychological cases. *Methods*. Participants were 15 patients who had a frontal lobe brain tumour around the insula cortex of the right hemisphere and had subsequently undergone a neurosurgical resective operation. Participants were asked to draw a human figure in both “hands-down” and “hands-up” configurations. *Results*. Eight of the 15 patients drew a human figure with six fingers during the “hands-up” and the “hands-down” human figure drawing tasks (one patient drew eight fingers). A statistical analysis of potential lesion areas revealed damage to the right anterior frontal insula and operculum in this group of patients relative to the five-finger drawing group. *Conclusions*. Our findings reveal a newly described neuropsychological phenomenon that could reflect impairment in attention directed towards body representations.

## 1. Introduction

**Figure 1:**
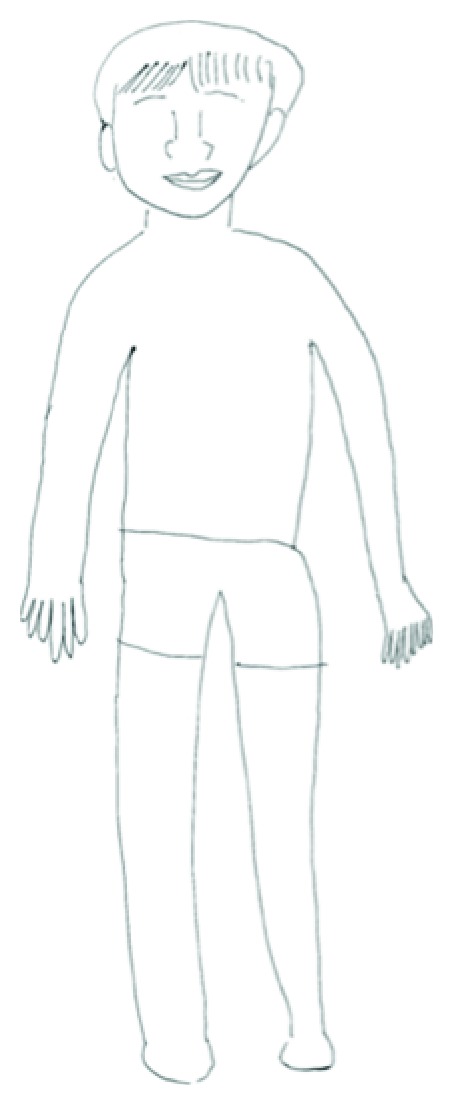
A human figure drawing by MM.

Perseveration, which involves inappropriate repetition of a response, is frequently observed in patients with brain damage. Perseveration can be observed across a wide range of behaviours, including writing, drawing, and speaking. Liepmann first systematically analysed the various types of perseveration [1], which led to a recent classification of three patterns of perseveration behaviour: (a) recurrent, that is, the repetition of the previous response to a subsequent stimulus, (b) stuck-in-set, that is, inappropriate maintenance of a given activity or type of response, and (c) continuous, that is, incorrect prolongation of a current behaviour [2, 3]. Type of perseveration may be related to the specific nature of the neurological impairment. Patients with aphasia produce significantly more recurrent perseveration than patients with damage to the right hemisphere or healthy controls. The stuck-in-set type of perseveration is found frequently in patients with Parkinson's disease, which suggests an association with dopamine system dysfunction. Continuous perseveration has been found in association with right hemisphere damage [3]. Several accounts have been proposed regarding causes of perseveration, including failure to inhibit a previous response [4], pathological inertia to a program of action [5], or attention deficits [3, 6, 7]. When we administered a human figure drawing test, a subtest of the behavioural inattention test (BIT) [8], to patient MM nine days after the resection of a brain tumour in the right insula and right frontal operculum, we found a previous unreported form of perseverative behaviour. She drew seven fingers on the figure's left hand and six fingers on the right (Figure 1); all other body parts were drawn correctly and she showed no perseverative performance for other behaviours such as writing or speaking. Prior to the resective operation, this patient drew five fingers on each hand during the same task. Following the operation, hemiparesis affected the left side of her body and her MMT score for the left arm was 0 or 1. She did not appear to suffer from anosognosia, somatoparaphrenia, and anosodiaphoria, given that she was very disappointed that her left side did not move after the operation. However, the MMT score for her left arm did improve to 1 or 2 after three months of rehabilitation. She showed good visual recognition of fingers and other body parts. Finger naming and pointing tasks involving her own and the tester's fingers were performed perfectly (each task, score of 10/10), and body part naming and pointing tasks (nose, ear, chin, hair, neck, shoulder, elbow, hand, knee, and leg) were also performed perfectly, for both her own and the tester's body (scores of 10/10 on each task). Despite these areas of preserved functioning, tactile recognition for the left side of the body was impaired. She was not able to provide correct responses for the fingers on her left hand (0/5), although she could recognize her left shoulder, upper and lower arms, knee, and elbow by tactile sensation. In addition, she showed no neurological signs of frontal lobe deficit (i.e., no deficits of inhibition). She could draw an octopus with eight tentacles from memory. She copied the correct number of petals for the flower on the BIT, a task that can elicit perseverative responses in other patients [9, 10]. Her counting ability was also intact. Her full score on the BIT was within the normal range (see Table 1). She did not show evidence of unilateral special neglect, given that she copied the left visual side of patterns perfectly. Therefore, it can be argued that her drawing of supernumerary fingers was not due to inattention to the outside world, deficits in visual constructive ability, deficits in counting ability, or deficits in semantic knowledge relating to body parts or body ownership. This “supernumerary finger drawing” phenomenon was also found again one month after the resection.

In the present study, we investigated whether the symptoms found in MM are commonly observed in patients with tumour resection of the insula and operculum of the right hemisphere. A hands-up human figure drawing task was devised for the present purpose of examining the number of fingers drawn by these patients. In our preliminary observations, some patients drew mitten-like hands without clearly separating the fingers during the more typical hands-down human figure task. We therefore devised an approach that implicitly forces patients to draw individual fingers, without giving them explicit instructions to do so. The hands-up human figure drawing task proved to be a useful method for eliciting drawings of individual fingers.

## 2. Method

### 2.1. Patients

Participants were 15 patients (12 females, 3 males; mean age = 36.9 years) who suffered from a frontal lobe brain tumour around the insula cortex of the right hemisphere and had subsequently undergone a neurosurgical resective operation. Two psychologists registered the patients for this study on the basis of preoperative MRI images. We collected data from 2004 to 2007. The characteristics and neuropsychological findings for all patients are shown in Table 1. All participants did not fail the immediately memory task of the Mini-Mental State Examination (MMSE) [11], suggesting that no patient struggled with concentration or arousal. Patients AMY and HM had orientation problems and hence may have had some problems of consciousness. Patient ST had a low MMSE score relative to the other patients (23/30), failing calculation and memory recall tasks, but her orientation was intact. Although there were three patients whose BIT scores fell under the cut-off score for significant impairment, these individuals could all draw whole objects during the copying and drawing tasks, such that no patient appeared to suffer from left-sided visual neglect. One patient (MN) was diagnosed with left-sided hemianopia. Perseverative behaviors in spatial cancellation tasks were not observed in all patients [10]. All participants gave their informed consent and were cooperative with the testing. All patients used their right hand for writing and drawing tasks. Lesions occurred at the nondominant hemisphere as determined by qualified brain surgeons. Five patients had left side hemiparalysis after operation. They were willing to participate in rehabilitation and therefore did not have anosognosia, somatoparaphrenia, or anosodiaphoria. MMT scores for these five patients just after their operations were 0-1 or 1-2, and after three month's rehabilitation, four patients out of five showed improvements in scores (MM's score was mentioned above, IM: 1-2, MN: 4, and HM: 4-5). Patients MA's hemiparalysis did not improve.

### 2.2. Task

Participants performed hands-down and hands-up versions of the human figure drawing task. The hands-down version was administered first in all cases. In addition to these two tasks, the MMSE and the BIT were administered (as described above). These tasks and neuropsychological tests were administered on the same day, at around two weeks postoperatively. The entire procedure took about 30 minutes. The human figure drawing tasks were also administered preoperatively for 11 of the 15 patients; this was not possible for the other four patients because their operations needed to happen on an urgent basis.

### 2.3. Neuroanatomical Analysis

Each patient's lesion area was mapped manually onto slices of a T1-weighted template MRI scan (Montreal Neurological Institute), using MRIcron software (http://www.mccauslandcenter.sc.edu/mricro/mricron/) [12]. The mapping was conducted by two brain surgeons who were blind to each patient's drawing task results; these surgeons also performed the resective surgeries for the participants in this study. The template was oriented to match the Talairach space [13]. Lesions were mapped onto slices that correspond to the *Z*-coordinated 40, 32, 24, 16, 8, 0, −8, −16, −24, and −32 mm Talairach coordinates. Since 8 patients underwent an urgent operation, preoperative DICOM data could not be acquired for semiautomatic MRIcron analysis. In lieu of this, the two neurosurgeons determined the location and boundaries of the lesions using pre- and postoperative T1 and T2 MR images. Statistical analysis of the resective stereotaxic maps was performed using MRIcron. For each voxel, patients were divided into two groups according to whether they did or did not draw extra fingers, and possible differences were analysed.

## 3. Results

### 3.1. “Six-Finger Drawing” Phenomena

**Figure 2:**
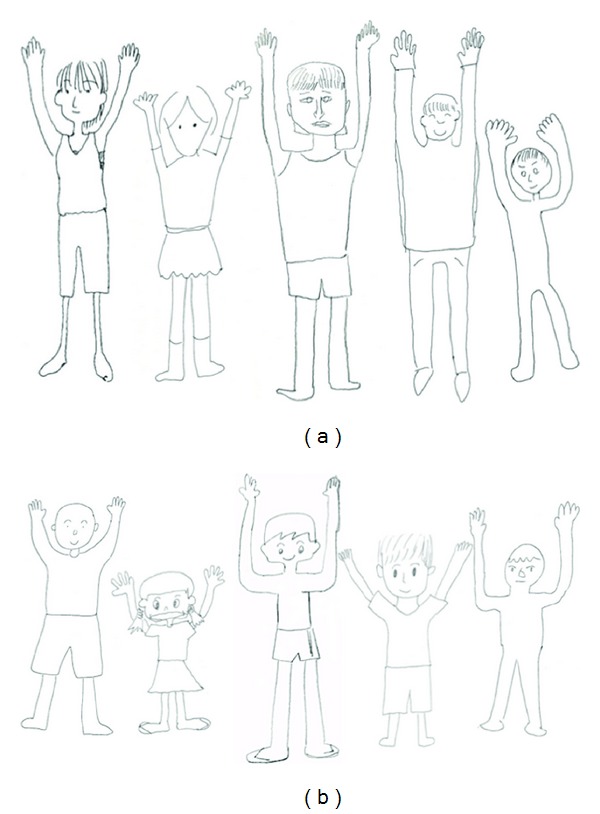
Examples of human figure drawings for each patient group. (a) Human figure drawings (hands-up) of five patients who drew six-fingered hands (from the left): HK, IM, MM, ST, and SY. (b) Human figure drawings (hands-up) of five-fingered hands (from the left): ANY, AY, HE, KH, and KM.

Of the 15 patients, eight (mean age = 37.5 years, SD = 9.3) drew a human figure with six-fingered hands during the hands-down and/or hands-up drawing tasks (the “six-finger drawing group,” which includes MM, who drew more than six fingers on one hand). The other 7 patients (mean age = 36.3 years, SD = 8.8) drew five-fingered hands during both tasks, the “five-finger drawing group.” Examples of the patients' drawings with supernumerary fingers are depicted in Figure 2(a), and patients' human figure drawings with normal fingers are depicted in Figure 2(b). Patients provided well-constructed finger drawings, although some patients did neglect to draw some parts of the face (nose or mouth). All other body parts were depicted in an appropriate manner. The average MMSE score for patients who drew supernumerary fingers was 27.9 (SD = 2.2) and 27.9 (SD = 1.6) for those who did not, which was not a statistically significant difference (*t*(12) = 0.0, ns).

### 3.2. Relevance of Paralysis to the Six-Finger Drawing Phenomenon

**Figure 3:**
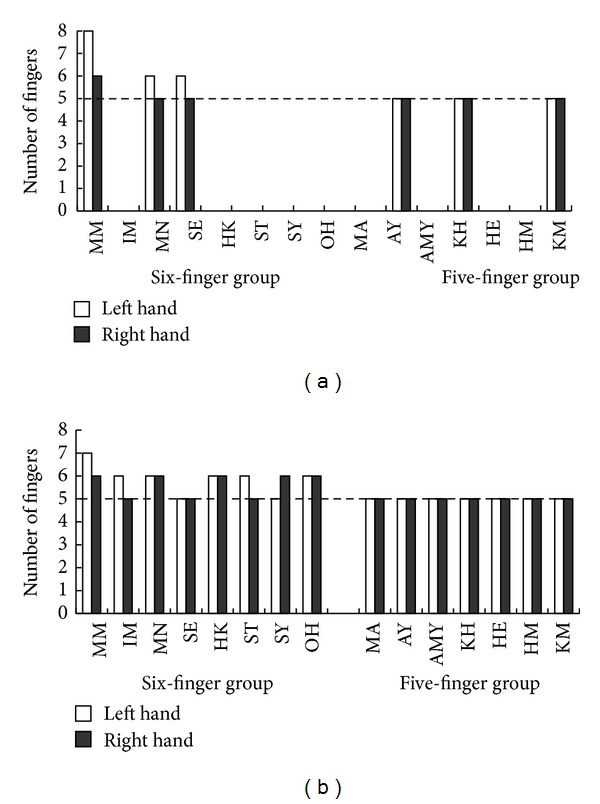
The number of fingers in each patient group. (a) The number of fingers for hands-down human figures drawn by each patient. Most of patients did not draw fingers in detail. (b) The number of fingers for hands-up human figures drawn by each patient.

The number of fingers for each limb posture drawn by each patient is shown in Figure 3(a) (hands-down task) and Figure 3(b) (hands-up task). For the six-finger drawing group, there was no significant difference between the number of fingers drawn on the right and left hands, both for the hands-up (*χ* ^2^(2) = 0.05, ns) and hands-down (*χ* ^2^(1) = 0.01, ns) drawing tasks. This suggests that there were no influences from the contralateral side of the lesion or having a paralyzed left hand on drawing the fingers. There were three patients with paralyzed limbs in the six-finger drawing group and two such patients in the five-finger drawing group, demonstrating that not all patients with paralyzed limbs produced six-finger drawings.

### 3.3. Preoperative Human Figure Drawing Task Data

Four of the eight patients who showed the six-finger drawing phenomenon provided drawings preoperatively. These patients did not draw six-fingered hands at this point. In the five-finger drawing group, all seven patients had performed the drawing task preoperatively; there were no patients who drew six-fingered hands.

### 3.4. Brain Lesion Analysis Related to the Six-Finger Drawing Phenomenon

**Figure 4:**
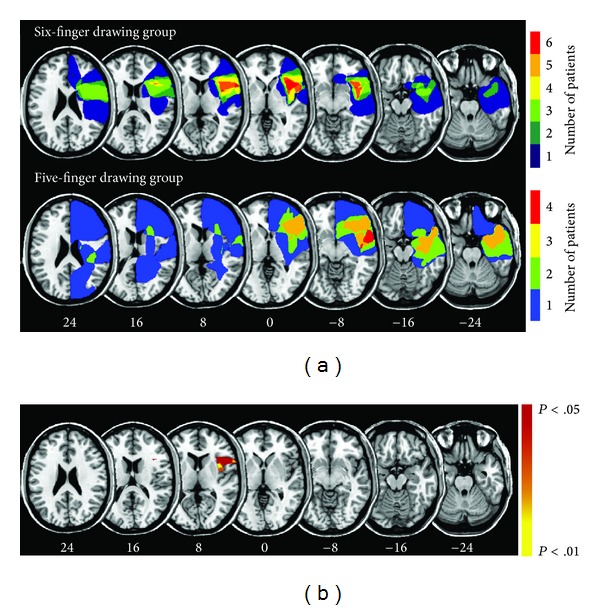
Lesion analysis for the six-finger drawing group. (a) Lesion overlap between six-finger and five-finger drawing groups (upper and middle images). (b) Brain areas revealed by statistical analysis as differing between the six-finger and five-finger drawing groups.

Brain lesions for each patient group were identified using a lesion-mapping tool, MRIcron. Both groups had lesions to the right frontal cortex, including the insula (Figure 4(a)). Fisher's test performed on the lesion density plots comparing the two patient groups revealed significant differences for the anterior insula and operculum (see Figure 4(b)). Damage to the right anterior insula and operculum appears to be related to the six-finger drawing phenomenon.

## 4. Discussion

To the best of our knowledge, this is the first systematic demonstration that supernumerary finger drawing is not a rare phenomenon but instead somewhat common and clearly linked to right anterior insula and operculum damage. All patient drawings studied here were well formed, suggesting no construction deficits. The six-finger drawing phenomenon was found not only in patients with paralyzed limbs but also in those without this paralysis. Limb paralysis does not appear to be related to the six-finger drawing phenomenon.

There are several possible interpretations of the six-finger drawing phenomenon. First, it could be argued that perseveration plays a role, since this deficit often occurs in cases of frontal lobe injury, particularly to the right lobe [5, 9, 14] including the Rolandic operculum and the insula [15]. The six-finger drawing phenomenon described here could be considered a “continuous” type of perseveration [2, 3]. Attention deficits provide convincing evidence for continuous perseveration [3, 6, 7]. Since attention is involved in various behaviours such as writing, speaking, and drawing, continuous perseveration usually manifests across a variety of tasks [16].

As described earlier, however, the patients in this study did not generally show perseveration in other behaviours. None of them manifested verbal perseveration behaviour and perseverative behaviour in cancellation tasks in BIT [10]. Although some patients drew supernumerary petals during the BIT, this was a relatively small number of patients across both six- and five-finger groups (two in the former group and one in the latter; see Table 1). General attention deficit is therefore not a particularly strong explanation of the six-finger drawing phenomenon, although attention deficits specific to body representation may provide a plausible account.

A second account of the six-finger drawing phenomenon is that it could reflect a deficit of hand representation or hemiparesis per se. Damage to the finger representations themselves could also play a role, although this interpretation alone does not explain why the patients drew a surplus of fingers. There were no patients in our sample who drew a smaller number of fingers. Furthermore, there were a relatively small number of patients with hemiparalysis in both the six-finger and five-finger drawing groups (see Table 1). It is therefore inconceivable that a hand representation deficit and hemiparalysis are a cause of the six-finger drawing phenomenon.

We did not explicitly instruct patients to draw individual fingers during our tasks. During the hands-down human figure drawing task, nine patients across the two groups did not draw detailed fingers but instead drew round or mitten-like hands. However, all patients drew detailed fingers during the hands-up task, irrespective of the number drawn. This suggests that participants attended and monitored body representations of the arms and hands (including fingers) regardless of conscious intent when drawing the hands-up human figure. It has been reported that endogenous attention to the body implicitly affects visual processing of the body [17], and repeated (perseverative) monitoring of such body representations could explain why our patients drew extra fingers. This account is supported by the observation that when the examiner directed patients' attention to the hands of the human figure drawing, patients were surprised that they drew too many fingers. A human drawing task that includes instructions to draw the fingers individually invites the risk that patients may realize the purpose of our examination. Once they realize this and pay careful attention, patients may in fact draw the correct number of fingers. However, not all of our patients spontaneously noticed the supernumerary fingers. The six-finger drawing phenomenon could reflect deficits of endogenous attention for body representations.

It has been argued that the right anterior insula cortex and operculum underlie a metarepresentation of the state of the body that is associated with subjective body awareness [18, 19]. The six-finger drawing phenomenon may reflect damage to such a system. We are aware of two additional patients, one with damage to the right frontal lobe and the other with damage to the left frontal lobe not involving the opercular and insular regions, who showed perseveration but not the six-finger drawing phenomenon. We have not experienced a patient who shows both perseverative responses and the six-finger phenomenon after frontal lobe damage not involving the opercular and insular regions. This suggests that right anterior insula cortex and operculum damage are specifically related to the six-finger phenomenon. This phenomenon did diminish a few months after the patients' resective operations. However, one year after the operation one of the patients who showed the six-finger drawing phenomenon, HK, noted that she sometime drops her bag when carrying it with her left hand, without intentional awareness of the hand in question. This experience may also reflect damage to an ongoing endogenous monitoring function with regard to body representation, which is considered to be one function of attention directed towards body representations.

## Figures and Tables

**Table 1:** Characteristics and neuropsychological results for patients in the present study.

Patient	Gender	Age	MMSE	BIT		Tumour grade	Hemiparalysis
				Total score^1^	Number of petals^2^		
Six-finger drawing group							
MM	F	37	**—**	140	9	III	+
IM	F	44	30	134	9	III	+
MN	F	43	28	87	9	III	+
SE	F	22	30	145	9	II	−
HK	F	24	27	142	9	II	−
ST	F	39	23	135	12	II	−
SY	M	40	28	143	9	III	−
OH	M	51	29	141	12	II	−

Five-finger drawing group							
MA	F	32	26	123	9	III	+
AY	F	33	30	146	9	II	−
AMY	F	26	27	114	8	IV	−
KH	F	40	28	141	9	III	−
HE	F	55	30	146	9	II	−
HM	M	38	26	143	10	III	+
KM	F	30	28	145	9	IV	−

^1^Full score for the BIT is 146 and the cut-off score is 131.

^2^Nine is the correct number of petals.
